# Euglycemic Diabetic Ketoacidosis-Like State During the Treatment of Diabetic Ketoacidosis in Fulminant Type 1 Diabetes: A Case Report

**DOI:** 10.7759/cureus.106472

**Published:** 2026-04-05

**Authors:** Keishi Yamauchi

**Affiliations:** 1 Diabetes and Endocrinology, International University of Health and Welfare Shioya Hospital, Yaita, JPN; 2 Internal Medicine, Asama Nanroku Komoro Medical Center, Komoro, JPN

**Keywords:** diabetic ketoacidosis (dka), euglycaemic diabetic ketoacidosis, fulminant type 1 diabetes mellitus, glucose supplementation, insulin deficiency

## Abstract

Diabetic ketoacidosis (DKA) is a life-threatening acute metabolic complication of diabetes characterized by hyperglycemia, metabolic acidosis, and ketosis. Appropriate insulin therapy and glucose supplementation during treatment are essential to prevent persistent ketoacidosis.

A 53-year-old man presented with a one-week history of polyuria. Urinalysis revealed 3+ ketonuria and 3+ glucosuria. Laboratory tests showed a hemoglobin A1c (HbA1c) level of 8.0%, plasma glucose at 720 mg/dL, arterial pH of 7.23, and bicarbonate (HCO₃⁻) at 15.9 mEq/L. He was diagnosed with DKA and was immediately admitted for treatment with intravenous saline and continuous regular insulin infusion. Eight hours after the initiation of therapy, plasma glucose decreased to 169 mg/dL; however, saline infusion without glucose supplementation was continued. After 30 hours, he was transferred to the diabetes department while still receiving saline and a minimal insulin infusion rate (0.1 U/h). At that time, despite a plasma glucose level of 125 mg/dL, metabolic acidosis persisted (pH 7.28) with continued 3+ ketonuria, consistent with persistent ketoacidosis despite euglycemia during treatment of DKA. The treatment regimen was revised to include glucose-containing intravenous fluids and an appropriate insulin dose. Following this adjustment, metabolic acidosis resolved. Further evaluation revealed serum C-peptide at <0.03 ng/mL and anti-glutamic acid decarboxylase (GAD) antibody at <5.0 U/mL, leading to a diagnosis of fulminant type 1 diabetes. This case highlights that insufficient glucose supplementation and inadequate insulin administration during DKA management may result in persistent ketoacidosis despite normalization of plasma glucose levels.

## Introduction

Diabetic ketoacidosis (DKA) is an acute metabolic complication characterized by hyperglycemia (plasma glucose >250 mg/dL), ketosis (elevated β-hydroxybutyrate levels), and metabolic acidosis (arterial blood pH ≤7.30 and bicarbonate [HCO₃⁻] ≤18 mEq/L). Without prompt treatment, DKA can progress to severe metabolic derangements and circulatory collapse, potentially resulting in death [1]. Although DKA is typically associated with marked hyperglycemia, it may also develop in patients with normal or only mildly elevated plasma glucose levels. Notably, the American Diabetes Association and the European Association for the Study of Diabetes do not define a strict plasma glucose threshold for the diagnosis of DKA [2,3]. DKA occurring at plasma glucose levels ≤250 mg/dL is termed euglycemic diabetic ketoacidosis (eDKA). This condition is characterized by the presence of ketoacidosis with relatively normal or mildly elevated blood glucose levels, which can delay diagnosis and appropriate management. In recent years, eDKA has been increasingly recognized as a potential adverse effect of sodium-glucose cotransporter 2 (SGLT2) inhibitors [4].

Here, we report a case of euglycemic diabetic ketoacidosis-like state that developed during DKA treatment, likely due to insufficient glucose supplementation.

The present case report was conducted in compliance with the Act on the Protection of Personal Information by fully anonymizing patient data. Written informed consent for publication was obtained from the patient. As this is a single case report, approval from an ethics committee was not required.

## Case presentation

A 53-year-old man presented with polyuria, polydipsia, and dry mouth that had persisted for approximately one week. His most recent health check, performed three months earlier, had shown normal blood glucose levels. On the day of presentation (day X), he visited the urology department of our hospital, where urinalysis revealed ketone bodies (3+) and glucose (3+). He was referred to the internal medicine department on the same day.

He reported consuming large amounts of water and tea but denied excessive intake of sugar-sweetened beverages. He had not experienced weight loss.

On admission, his vital signs were as follows: height, 170 cm; weight, 70 kg; temperature, 36.8°C; blood pressure, 112/72 mmHg; pulse, 70 beats/min; and respiratory rate, 16 breaths/min. He was fully alert. Laboratory results revealed a hemoglobin A1c (HbA1c) level of 8.0% and a plasma glucose level of 720 mg/dL. Arterial blood gas analysis showed a pH of 7.23, bicarbonate (HCO₃⁻) at 15.9 mEq/L, and pCO₂ at 15.8 mmHg. Serum ketone analysis demonstrated the acetoacetate value of 758 μmol/L and β-hydroxybutyrate at 31,493 μmol/L, consistent with DKA based on metabolic acidosis and elevated serum ketone levels (Table 1). A diagnosis of DKA was made, and the patient was admitted to the hospital.

**Table 1:** Laboratory data on admission. The patient presented with marked hyperglycemia, ketosis, and metabolic acidosis. Serum osmolality was within the normal range despite severe metabolic derangements. Abbreviations: HbA1c, hemoglobin A1c; IRI, immunoreactive insulin; CPR, C-peptide reactivity; Anti-GAD antibody, anti–glutamic acid decarboxylase antibody; IA-2, insulinoma-associated antigen-2; WBC, white blood cells; RBC, red blood cells; Hb, hemoglobin; Ht, hematocrit; Plt, platelets; TP, total protein; Alb, albumin; AST, aspartate aminotransferase; ALT, alanine aminotransferase; LDH, lactate dehydrogenase; CK, creatine kinase; UN, urea nitrogen; Cr, creatinine; CRP, C-reactive protein.

Category	Parameter	Value	Reference range
Diabetes-related markers	Plasma glucose	720 mg/dL	70-109 mg/dL
	HbA1c	8.0%	4.9-6.0%
	IRI	1.02 μU/mL	2-10 μU/mL
	CPR	0.05 ng/mL	0.6-2.1 ng/mL
Autoimmune markers	Anti-GAD antibody	<5 U/mL	<5 ng/mL
	Anti-insulin antibody	<0.4 U/mL	<0.4 U/mL
	Anti-IA-2 antibody	<0.6 U/mL	<0.6 U/mL
Acid–base status	pH	7.231	7.35-7.45
	pCO₂	15.8 mmHg	35-48 mmHg
	HCO₃⁻	15.9 mEq/L	24-31 mEq/L
	Anion gap	16 mEq/L	8-12 mEq/L
Ketone bodies	β-hydroxybutyrate	3493 μmol/L	<85 μmol/L
	Acetoacetate	758 μmol/L	<55 μmol/L
Osmolality	Plasma osmolality	301 mOsm/kg H₂O	275-295 mOsm/kg H₂O
Electrolytes	Na	128 mEq/L	138-145 mEq/L
	Corrected Na	138 mEq/L	138-145 mEq/L
	K	5.1 mEq/L	3.6–4.8 mEq/L
	Cl	106 mEq/L	98–108 mEq/L
Urinalysis	pH	5.0	5.0–7.5
	Protein	−	−
	Occult blood	−	−
	Sugar	3+	−
	Ketones	3+	−
Blood cell count	WBC	7480/μL	3300-8600/μL
	Neutrophils	52.0%	40-70%
	RBC	5.18 ×10⁶/μL	4.3-5.7/μL
	Hb	14.7 g/dL	13.7-16.8 g/dL
	Ht	45.3%	40-50%
	Plt	1.62×10⁵/μL	16-35/μL
Biochemistry	TP	7.8 g/dL	6.5-8.2 g/dL
	Alb	4.4 g/dL	3.7-5.5 g/dL
	AST	18 U/L	10-40 U/L
	ALT	18 U/L	5-45 U/L
	LDH	147 U/L	120–245 U/L
	Amylase	56 U/L	39-134 U/L
	CK	254 U/L	50-230 U/L
	UN	20.2 mg/dL	8-20 mg/dL
	Cr	0.98 mg/dL	0.6-1.1 mg/dL
	CRP	0.2 mg/dL	<0.3 mg/dL

Hospital course

**Figure 1:**
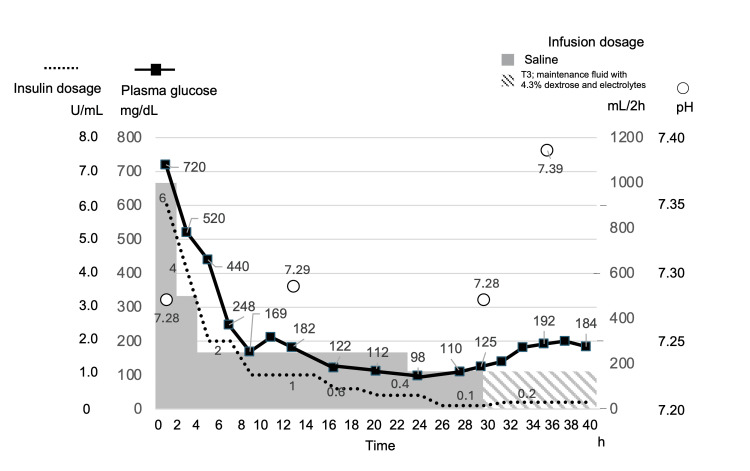
Clinical course showing changes in plasma glucose, pH, insulin infusion rate, and fluid administration Plasma glucose levels improved with saline and insulin administration; however, metabolic acidosis persisted despite glycemic correction. Subsequent glucose supplementation led to an improvement in acidosis.

Initial management included approximately 4 L of intravenous normal saline over 24 hours and continuous intravenous insulin infusion at 6 units/h (Figure 1). Plasma glucose declined rapidly, reaching 440 mg/dL after 4 hours and 169 mg/dL after 8 hours. Despite this decrease, normal saline infusion was continued.

After 24 hours, the insulin infusion rate was reduced to 0.4 units/h as plasma glucose stabilized at approximately 90 mg/dL and was further reduced to 0.1 units/h.

At 30 hours, the patient was transferred to the Department of Diabetes, Metabolism, and Endocrinology. At that time, plasma glucose was 125 mg/dL; however, metabolic acidosis persisted (pH 7.28, HCO₃⁻ 15.0 mEq/L, urinary ketones 3+, β-hydroxybutyrate 3,323 μmol/L). Serum magnesium, phosphorus, and calcium levels were 1.8 mg/dL, 2.0 mg/dL, and 8.6 mg/dL, respectively. These findings were consistent with persistent ketoacidosis despite euglycemia during treatment of diabetic ketoacidosis.

Intravenous fluid therapy was initially performed using glucose-free fluids. Subsequently, the infusion was switched to Solita T3 solution (Yoshindo Inc., Toyama, Japan), which contains both glucose and potassium (21.5 g of glucose per 500 mL). At an infusion rate of 100 mL/h, this corresponded to approximately 4.3 g/h of glucose administration. The insulin infusion rate was subsequently adjusted. Six hours later, the arterial pH normalized to 7.38, and urinary ketones disappeared by the following day.

Additional investigations revealed a fasting serum C-peptide level of <0.03 ng/mL, undetectable urinary C-peptide, negative anti-glutamic acid decarboxylase (GAD) and anti-insulinoma-associated antigen-2 (IA-2) antibodies, and absent insulin secretion on glucagon stimulation testing. Pancreatic enzyme levels were normal. Based on these combined findings, including absent insulin secretion on glucagon stimulation and negative islet-related autoantibodies, the patient was diagnosed with fulminant type 1 diabetes. After resolution of ketoacidosis, multiple daily insulin therapy with basal and bolus regimens was initiated, and the patient was subsequently discharged.

## Discussion

Although hyperglycemia often resolves rapidly with insulin therapy, correction of metabolic acidosis in DKA requires a longer time course. β-Hydroxybutyrate, the predominant ketone, may take more than 24 hours to normalize in severe cases [5]. While insulin promptly suppresses new ketone production, clearance of preexisting ketone bodies is delayed.

DKA is driven by absolute insulin deficiency, resulting in unopposed secretion of counter-regulatory hormones such as glucagon, cortisol, and catecholamines [1]. This hormonal imbalance enhances hepatic gluconeogenesis and glycogenolysis, reduces peripheral glucose utilization, and stimulates lipolysis. Free fatty acids are mobilized and converted into ketone bodies, which are the primary cause of metabolic acidosis. Osmotic diuresis induced by hyperglycemia further exacerbates dehydration and hypovolemia. In addition, continued administration of chloride-rich saline may contribute to hyperchloremic metabolic acidosis [6].

By contrast, eDKA arises primarily from carbohydrate depletion [7]. In this state, insulin deficiency is relatively mild, but the glucagon-to-insulin ratio remains elevated, driving hepatic ketogenesis with limited effects on glucose metabolism [8,9]. Fasting, prolonged exercise, or depletion of hepatic glycogen stores can promote ketone body production via acetyl-CoA (coenzyme A) metabolism [10]. This mechanism is also implicated in SGLT2 inhibitor-associated eDKA [11].

During DKA treatment, clinical guidelines recommend transitioning from saline to glucose-containing fluids once plasma glucose falls below 250 mg/dL [12]. According to the 2024 American Diabetes Association guidelines [13], dextrose-containing fluids should be initiated when plasma glucose falls to 200-250 mg/dL to maintain levels between 150 and 200 mg/dL until resolution of DKA. While fluid resuscitation and electrolyte replacement are essential in the initial phase of DKA, adequate glucose supplementation becomes important in the later phase to allow continued insulin administration and resolution of ketosis. However, the rationale for this recommendation is often insufficiently emphasized. In the present case, continued administration of normal saline after normalization of plasma glucose deprived the patient of an adequate carbohydrate supply, impairing ketone clearance despite ongoing insulin therapy and ultimately resulting in eDKA. Concomitant hyperchloremia may also have contributed to the persistence of metabolic acidosis. This case highlights the importance of adequate glucose supplementation during DKA management to ensure continued insulin administration and effective clearance of ketone bodies.

## Conclusions

This case highlights that during the management of DKA, insufficient glucose supplementation can precipitate euglycemic ketoacidosis despite normalization of plasma glucose levels. Timely initiation of glucose-containing intravenous fluids, in parallel with ongoing insulin therapy, is essential to facilitate ketone clearance and resolve metabolic acidosis. Clinicians should be aware that inadequate adjustment of glucose and insulin therapy during treatment may lead to persistent ketoacidosis even when plasma glucose levels appear controlled. Careful monitoring of acid-base status and ketone levels is therefore crucial in the management of DKA.
